# Giant borderline ovarian tumours – review of the literature

**DOI:** 10.1515/med-2025-1267

**Published:** 2025-08-19

**Authors:** Pawel Sadlecki, Katarzyna Dejewska, Patrycja Domieracka, Malgorzata Walentowicz-Sadlecka

**Affiliations:** Faculty of Medicine, University of Science and Technology, Bydgoszcz, Poland; Department of Obstetrics, Gynecology and Gynecologic Oncology, Regional Polyclinical Hospital, Grudziadz, Poland; Department of Obstetrics, Gynecology and Gynecologic Oncology, Regional Polyclinical Hospital, Torun, Poland

**Keywords:** giant borderline ovarian tumour, surgical treatment, giant, borderline, borderline malignancy, ovarian tumour, ovarian cyst

## Abstract

**Introduction:**

Giant borderline ovarian tumours (GBOTs) are rare neoplasms that require meticulous management to prevent high-risk operative complications. The broader goal of this systematic review is to consolidate the existing knowledge on GBOTs by focusing on diagnostic approaches, differential diagnoses, and treatment strategies. Furthermore, the relationship between the clinical features of GBOTs and the types of diagnostic and therapeutic procedures implemented was determined.

**Materials and methods:**

The publications were analysed for the following data: histopathological type of GBOT; patient’s age; dimensions, weight, and/or volume of the tumour; levels and types of tumour markers determined; types of imaging tests performed; type of treatment applied.

**Results:**

Twenty-one articles describing the clinical situation of 22 patients met the inclusion criteria for the systematic review. The mean age of the patients included in the analysis was 46.68 years (SD: 19.1 years); the youngest patient was 12, and the oldest was 76 years of age. In the analysed literature, patients most often (81.8%) had the mucinous type of GBOT. In the vast majority of cases (86.36%), based on the analysed literature, the surgical treatment method for the patients was laparotomy. In more than half of the patients (54.55%), the uterus was removed during surgical treatment. In the analysed literature, the hysterectomy procedure was not performed in patients under 40 years of age. Based on the analysed literature, it was found that if the CA 125 concentration in the blood serum of patients with mucosal tumours exceeded 40 U/mL, laparoscopy was not performed and the patients were treated using an open approach.

**Conclusions:**

GBOTs are rare neoplasms that require meticulous management to prevent high-risk operative complications. Despite the diagnostic and therapeutic challenges posed by the large size and potential complications of these tumours, with proper medical care, patients can achieve successful outcomes and a good prognosis.

## 1 Introduction

In an era of growing popularity of minimally invasive techniques in surgical gynaecology, giant borderline ovarian tumours (GBOTs) remain a niche in which classical surgical skills will undoubtedly remain essential. GBOTs are defined as ovarian masses of at least 20 cm in diameter. These tumours are relatively rare in the existing medical literature and present with a wide range of clinical manifestations [1]. BOTs typically occur in women who are about a decade younger than those diagnosed with epithelial ovarian cancer, with the majority of cases (approximately 75%) being detected at an early stage, particularly stage I, according to the International Federation of Gynaecology and Obstetrics (FIGO) [2]. GBOTs are characterised by slow growth and carry the potential to transform into malignant ovarian tumours [3]. Their large size and associated complications present unique diagnostic and therapeutic challenges, although advancements in imaging and healthcare have made the occurrence of such massive tumours increasingly rare [4]. The symptoms of GBOTs are similar to those of other ovarian tumours, including abdominal pain, bloating, and irregular menstrual cycles. Additionally, compressive symptoms or a visible abdominal mass is commonly observed [2]. Due to their size, these tumours can cause significant discomfort and hinder daily activities. The primary diagnostic tools are imaging techniques such as ultrasound (USG), computed tomography (CT), or magnetic resonance imaging (MRI). Diagnostics also include the evaluation of tumour markers. Surgical treatment generally consists of laparotomy with varying extents of organ removal, depending on the individual case [1].

The aim of this systematic review is to compile and evaluate the current knowledge on GBOTs, with particular focus on diagnostic methods, differential diagnosis, and treatment strategies. We emphasise the importance of preoperative assessment and a critical evaluation of surgical options. Although open laparotomy has traditionally been the standard approach – especially given the massive size of these tumours – minimally invasive techniques may be considered in a few carefully selected cases. Consequently, this review explores both the evolving role of laparoscopy and other minimally invasive methods, as well as the full range of surgical interventions available for managing large ovarian masses.

## 2 Materials and methods

**Figure 1:**
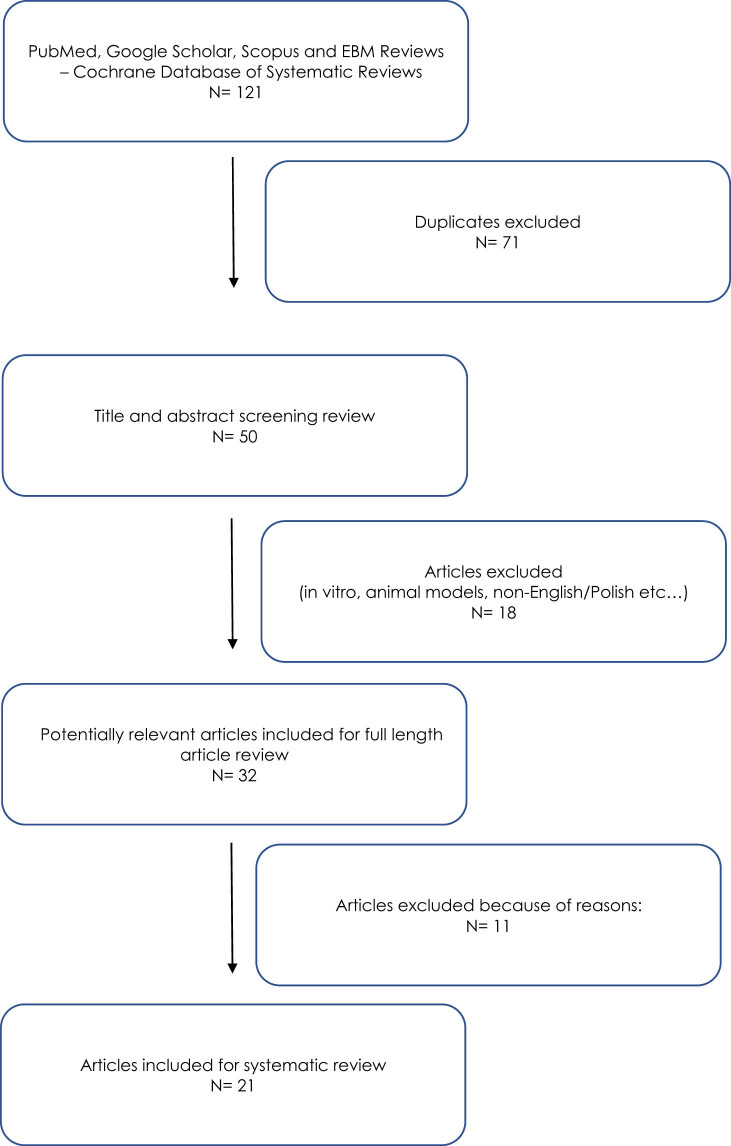
Flow diagram of the study selection process.

This systematic review was conducted in accordance with the international standards and guidelines for systematic reviews (PRISMA – Preferred Reporting Items for Systematic Reviews and Meta-Analyses). The PRISMA 2020 checklist was applied in this study. A detailed review protocol is available from the author upon request. The review included publications from databases such as PubMed, Google Scholar, Scopus, and EBM Reviews (including the Cochrane Database of Systematic Reviews), covering articles published between 2000 and 2024. To increase precision, we incorporated Boolean operators (AND/OR) into our search strategy and used a targeted combination of keywords – “giant and borderline,” “borderline malignancy,” “ovarian tumour,” and “ovarian cyst” – to identify eligible studies. To ensure transparency and minimise selection bias, study selection, data extraction, and quality assessment were conducted independently by two reviewers; any disagreements were resolved through discussion and voting on disputed articles. If, following discussion, the reviewers reached unanimous agreement, the publication was included in the analysis. Searches were conducted on August 11, 2024. The Newcastle–Ottawa Scale was implemented to assess the quality of the included studies. The review was limited to publications in English or Polish and excluded repeated items and articles without full-text availability. The inclusion of publications in English and Polish was justified by the authors’ proficiency in these languages, which allowed for a thorough analysis of the content. The initial analysis primarily included peer-reviewed case reports, observational studies, and retrospective analyses. A secondary search entailed examining the reference lists of all the included articles. Certain publication types, such as editorials, comments, conference abstracts, abstracts, validation studies, and animal studies, were excluded from the analysis. Studies were excluded from the review if the diagnosis was other than a borderline tumour; the tumour size was less than 19 cm, weighed less than 1,000 g, or had a volume less than 500 mL; or the clinical data or results of the pathological examination were not reported. The inclusion and exclusion criteria for this study are summarised in Table 1, and a flow diagram illustrating the study selection process is presented in Figure 1. The publications were analysed for the following data: histopathological type of GBOT; patient’s age; dimensions, weight, and/or volume of the tumour; levels and types of tumour markers determined; types of imaging tests performed; and type of treatment applied.

**Table 1:** Inclusion and exclusion criteria for the study

**Inclusion criteria**	
Type of study	Peer-reviewed: Case reports, case series, and observational studies
Years	2000–2024
Language	English and Polish
Subject of study	Live humans
Clinical criteria	Borderline ovarian tumour (pathologically confirmed) and reported clinical data
**Exclusion criteria**	
Type of study	Editorials, comments, conference abstracts, abstracts, book chapters, and validation studies
Language	Other than English and Polish
Subject of study	Animals and autopsy
Clinical criteria	Size <20 cm or weight <1,000 g or volume <500 mL, not reported: Clinical data or pathological confirmation

Statistical analyses were performed using the PQStat statistical package version 1.8.4.152. The scale results were compared using the Mann–Whitney U test, and a logistic regression model was also estimated. A test probability of *p* < 0.05 was considered significant, while a value of *p* < 0.01 was considered highly significant.

## 3 Results

After the first search of the databases (PubMed, Google Scholar, Scopus, and EBM Reviews, which included the Cochrane Database of Systematic Reviews) for the given keywords, 121 items were obtained. Duplicates were then excluded, leaving 50 publications. After the analysis of the abstracts of the selected publications, 32 items qualified for further analysis. Due to the lack of criteria allowing for qualification for the systematic review, another 11 publications were eliminated. Finally, 21 articles describing the clinical situation of 22 patients met the inclusion criteria for the systematic review. The clinical data obtained from the publications included in the systematic review are summarised in Table 2.

**Table 2:** Clinical data obtained from publications included in the systematic review (BOT – borderline ovarian tumour, HA – abdominal hysterectomy, TLH – total laparoscopic hysterectomy, BSO – bilateral salpingo-oophorectomy, USO – unilateral salpingo-oophorectomy, CT – computed tomography, MRI – magnetic resonance imaging, USG – ultrasound examination, CA 125 – cancer antigen 125, CA 19-9 – cancer antigen 19-9, CEA – carcinoembryonic antigen, AFP – α-fetoprotein, ß-hCG – ß-human chorionic gonadotropin, and ND – no data)

	Author	No. of BOT CASES	Histological type of BOT	Max. dimensions/weight/volume	Age	Symptoms	Tumour markers	Diagnostics	Surgical treatment
1	Mikos T	1	Mucinous/serous	35 l	59	Dyspnoea abdominal distension	CA 125, 300 U/mL	USG and CT	Laparotomy and HA + BSO
2	Iwasaki M	1	Mucinous	21 × 15.5 × 7 cm/1,120 g	13	Abdominal pain, abdominal distension, abdominal girth, and fever	CA 125, 184.0 U/mL; CA 19-9, 330.0 U/mL	USG and CT	Laparotomy, USO, and appendectomy
3	Cîrstoiu MM	1	Mucinous	59 × 48 × 32 cm/30 kg	44	Abdominal pain, abdominal distension, constipation, and early satiety	CA 125, CA 19-9, CEA, and AFP – normal values	USG and CT	Laparotomy and HA + BSO
4	Lee HM	1	Mucinous	19 × 15 × 8.5 cm/1,584 g	13	Abdominal distension	CA 125, 284 U/mL; CA 19-9, 2,581 U/mL	PET/CT	Laparotomy, unilateral USO, appendectomy, and omentectomy
5	Dougherty D	1	Mucinous/Brenner	40 × 22 × 27 cm	57	Abdominal discomfort	CA 125, 249.4 U/mL; CEA, 6.1 ng/mL	CT	Laparotomy and HA + BSO
6	Pilone V	1	Mucinous	60 × 50 × 40 cm/6,500 g (solid part)	69	Abdominal distension	CA 125, CEA, AFP, and CA 19-9 – normal values	CT	Laparotomy, HA + BSO, and partial omentectomy
7	Watanabe S	1	Mucinous	42 × 22 × 20 cm/11,800 g	12	Abdominal distention and abdominal girth	CA 125, 96.3 U/mL; CEA, 117.5 ng/mL; AFP, 0.7 ng/mL; CA 19-9, 5029.0 U/mL; ß-hCG <0.003 units/mL	USG, MRI, and X-ray	Laparotomy and USO
8	Yazawa H	1	Endometrioid	27 × 9 cm	41	Abdominal/back pain	CA 125, 150 U/mL; CA 19‐9, 220 IU/mL	MRI	Laparoscopy, TLH + BSO, partial omentectomy, and pelvic lymph node biopsy
9	Mulita F	1	Mucinous	44 × 39 × 19 cm/15.4 kg	59	Abdominal distention, dyspnoea, and difficulty in ambulation	CA 19-9, >953 U/mL	ND	Laparotomy, HA + BSO, omentectomy, and appendectomy
10	Mitragkas P	2	1. Mucinous; 2. mucinous	1. 23.5 × 11 × 23.6 cm; 2. 24,7 cm	1. 45; 2. 54	1. Constipation and early satiety; 2. Abdominal bloating and epigastrium pain	1. All tumour markers were in normal range; 2. All tumour markers were in normal range	1. USG and CT; 2. USG	1. Laparotomy and USO; 2. Laparoscopy, TLH + BSO, and omentectomy
11	Deo A	1	Mucinous	36 × 30 × 18 cm/32.5 kg	76	Massive ascites and breathlessness (NYHA III), abdominal distension, abdominal pain, weight loss, and constipation	CA 125, 53 U/mL; CA 19-9, 1,000 U/mL; CEA; ß-HCG; AFP – normal	USG and CT	Laparotomy – HA + BSO, biopsy (vaginal cuff, peritoneum, and omentum)
12	Halani D	1	Mucinous	35 × 40 × 32 cm/24 kg	53	Abdominal pain	CA 125, 53.2 IU/mL; CA 19-9, 1,000 U/mL	CT	Laparotomy and HA + BSO
13	Yazawa R	1	Mucinous	>20 cm	26	ND	CA 125, 55.7 U/mL; CA 19-9, 177 U/mL; CEA, 16.6 ng/mL	CT and MRI	Laparotomy, USO, and appendectomy
14	Peiretti M	1	Mucinous	47 × 36 × 33 cm	52	Abdominal distention, reflux, early satiety, constipation, difficulty in ambulation, and dyspnoea	CA 125, 33 U/mL; CEA, 0.97 ng/mL; CA 19-9, 19.2 U/mL; AFP, 1.1 ng/mL	USG and CT	Laparotomy, HA + BSO, intraoperative controlled drainage appendectomy, and abdominal wall reconstruction
15	Gharbia N	1	Mucinous	27 × 12 × 26 cm	30	Abdominal pain and abdominal girth over the past fever	CA 125, 493 U/mL; CA 19-9, 273 U/mL	USG and MRI	Laparotomy, USO, omentectomy, and appendectomy
16	Onuzo CN	1	Mucinous	50 cm	24	Abdominal discomfort	CA 125, 54.3 U/mL	USG and MRI	Laparotomy and USO
17	Bogliatto F	1	Mucinous	34 cm	60	Abdominal pain	CA 125, CEA, AFP, and CA 19-9 – normal levels	USG and CT	Laparoscopy, intraoperative controlled drainage, and BSO
18	Stukan M	1	Mucinous	50 × 45 × 26 cm	49	ND	CA 125, 140.4 U/mL	CT	Laparotomy and HA + BSO
19	Berbecar VT	1	Mucinous	19 × 19 × 31 cm	59	Abdominal discomfort	ND	USG and CT	Laparotomy and USO
20	Rigo F	1	Serous	26 × 33 × 20 cm/10 kg	69	Abdominal distension	CEA, 5.2 ng/mL; CA 19-9, 103 IU/mL; AFP, 0.8 IU/mL	CT	Laparotomy, HA + BSO, and appendectomy
21	Pence S	1	Mucinous	35 × 25 × 35 cm	63	Septic shock	ND	ND	Laparotomy, BSO, and reparation of peptic ulcers

The mean age of the patients included in the analysis was 46.68 years (SD 19.1 years); the youngest patient was 12 years of age, and the oldest was 76 years of age. In the analysed literature, patients most often (81.8%) had the mucinous type of GBOT. Moreover, patients up to 40 years of age had only the mucinous type of BOT. The tumours were analysed in terms of the largest dimension; the mean value was 36.16 cm, and the median was 35 cm. Half of the tumours were in the range of 27–44 cm, while the total range of results was 19–60 cm. The relationship between the maximum tumour size and age was examined, but no statistically significant relationship was found. In the analysed publications, the average tumour weight was 14,767 g, and the median was 11,800 g; the lightest tumour weighed was 1,120 g, and the heaviest was 32,500 g. All tumours in the cases analysed were limited to a single ovary. The average CA 125 level in the blood was 126.9 U/mL, with a median of 55 U/mL, and values ranged from 25 to 493 U/mL. We also looked at how CA 125 is related to age and found a nearly significant pattern (*p* = 0.0546): we observed a trend toward higher average and median CA 125 levels in patients  ≤40 years (*p*  =  0.0546) (Table 3). Based on the analysed literature, it was found that if the CA 125 concentration in the blood serum of patients with mucinous borderline tumours exceeded 40 U/mL, laparoscopy was not performed, and the patients were treated using an open approach. In the vast majority of cases (86.36%), based on the analysed literature, the surgical treatment method for the patients was laparotomy. In more than half of the patients (54.55%), the uterus was removed during surgical treatment. In the analysed literature, the hysterectomy procedure was not performed in patients under 40 years of age.

**Table 3:** Relationship between the CA 125 concentration in blood serum and age; a relationship close to statistical significance was found (*p* = 0.0546) (SD – standard deviation, Q1 – first quartile, and Q3 – third quartile)

		CA 125 (U/mL)	
		≤40 (years)	>40 (years)
Mean		194.55	93.0833
SD		170.7556	95.8221
Median		140.15	43
Minimum		54.3	25
Maximum		493	300
Q1		65.85	29.5
Q3		259	142.8
Mann–Whitney U test	*Z*	1.9220	
	*p*	0.0546	

**Figure 2:**
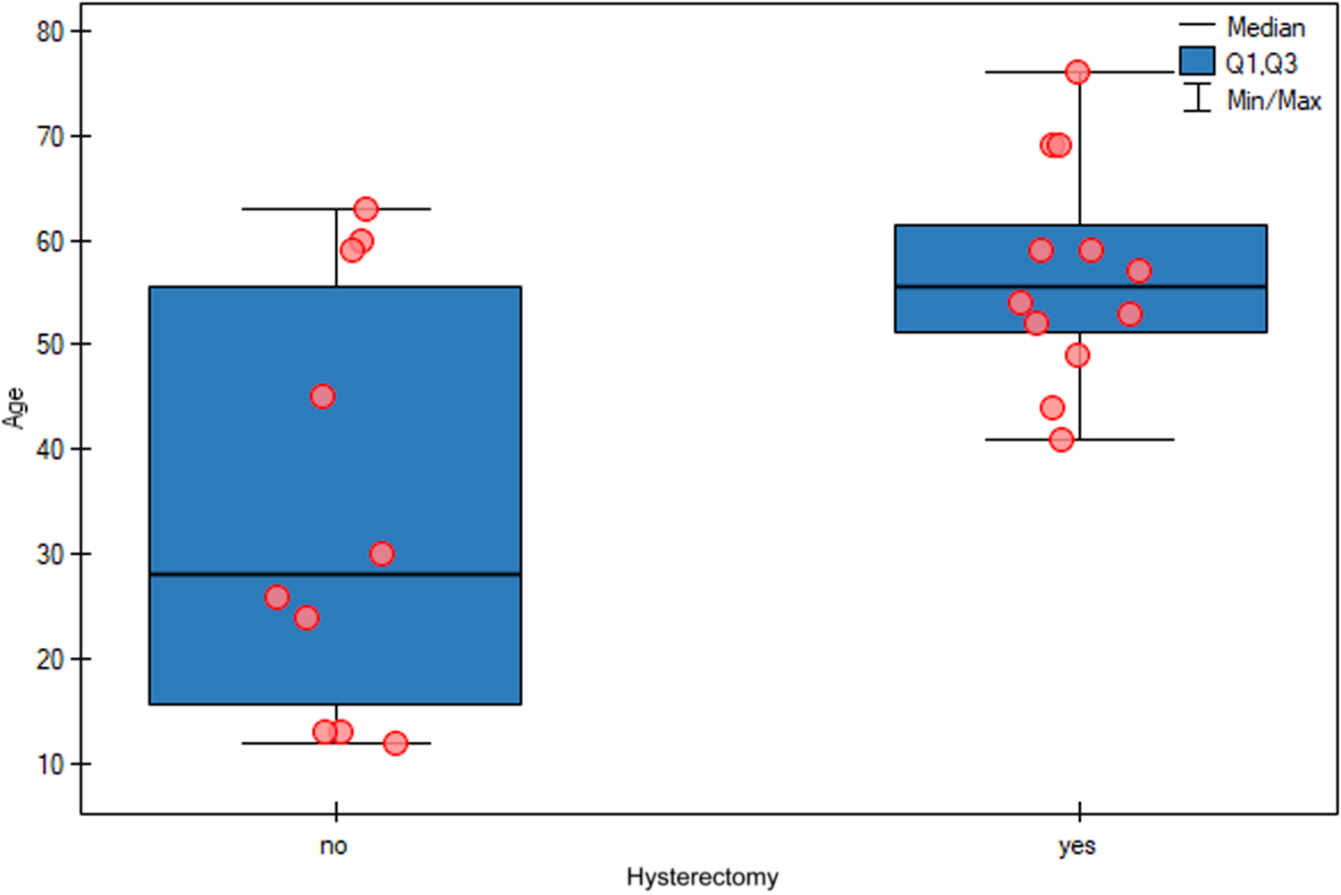
Relationship between age and the decision to perform hysterectomy. In older patients, hysterectomy was statistically significantly more often performed (*p* = 0.0375).

**Figure 3:**
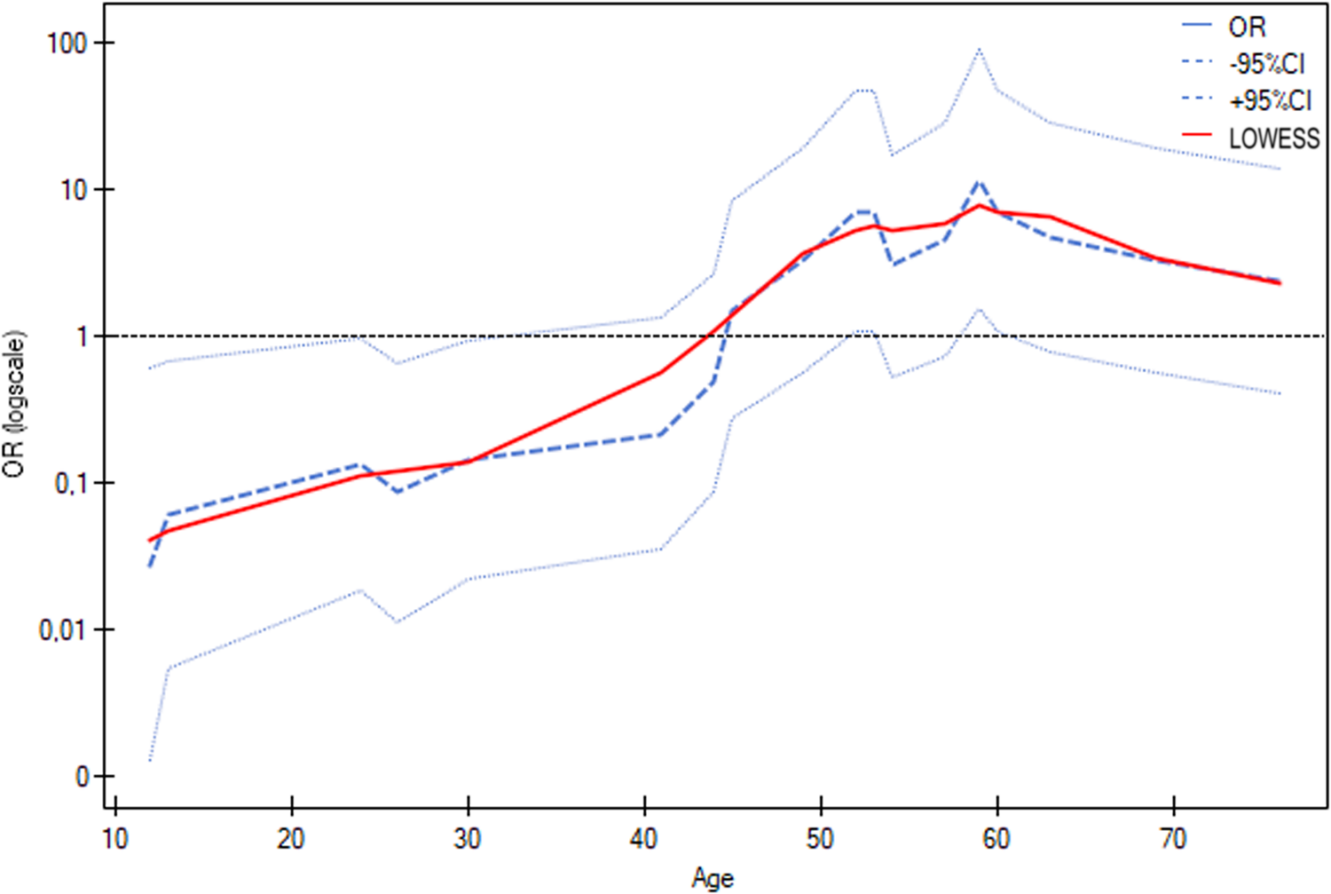
Logistic regression model of the prediction of hysterectomy by age indicates a significant effect of age, as the odds ratio is 1.0923 with a 95% confidence interval from 1.0127 to 1.1781 (*p* = 0.0222).

The relationship between age and the decision to perform hysterectomy was also examined, and in older patients, hysterectomy was statistically significantly more often performed (*p* = 0.0375) (Table 4 and Figure 2). The logistic regression model for the prediction of hysterectomy by age indicated a significant effect of age, as the odds ratio was 1.0923, with a 95% confidence interval from 1.0127 to 1.1781 (*p* = 0.0222). This means that each additional year of age was associated with a higher probability of undergoing hysterectomy, confirming a statistically significant effect of age (Figure 3). In five instances, fertility-sparing treatment was chosen due to the patient’s age. Appendectomy was performed in seven cases. The reviewed literature mostly did not report the duration of surgery or intraoperative blood loss.

**Table 4:** Age in the group of the patients with and without hysterectomy

		Age (years)	
		Without hysterectomy	With hysterectomy
Mean		34.5	56.8333
SD		20.5656	10.4258
Median		28	55.5
Minimum		12	41
Maximum		63	76
Q1		15.75	51.25
Q3		55.5	61.5
Mann–Whitney U test	*Z*	2.0806	
	*p*	0.0375	

(SD – standard deviation, Q1 – first quartile, and Q3 – third quartile).

**Figure 4:**
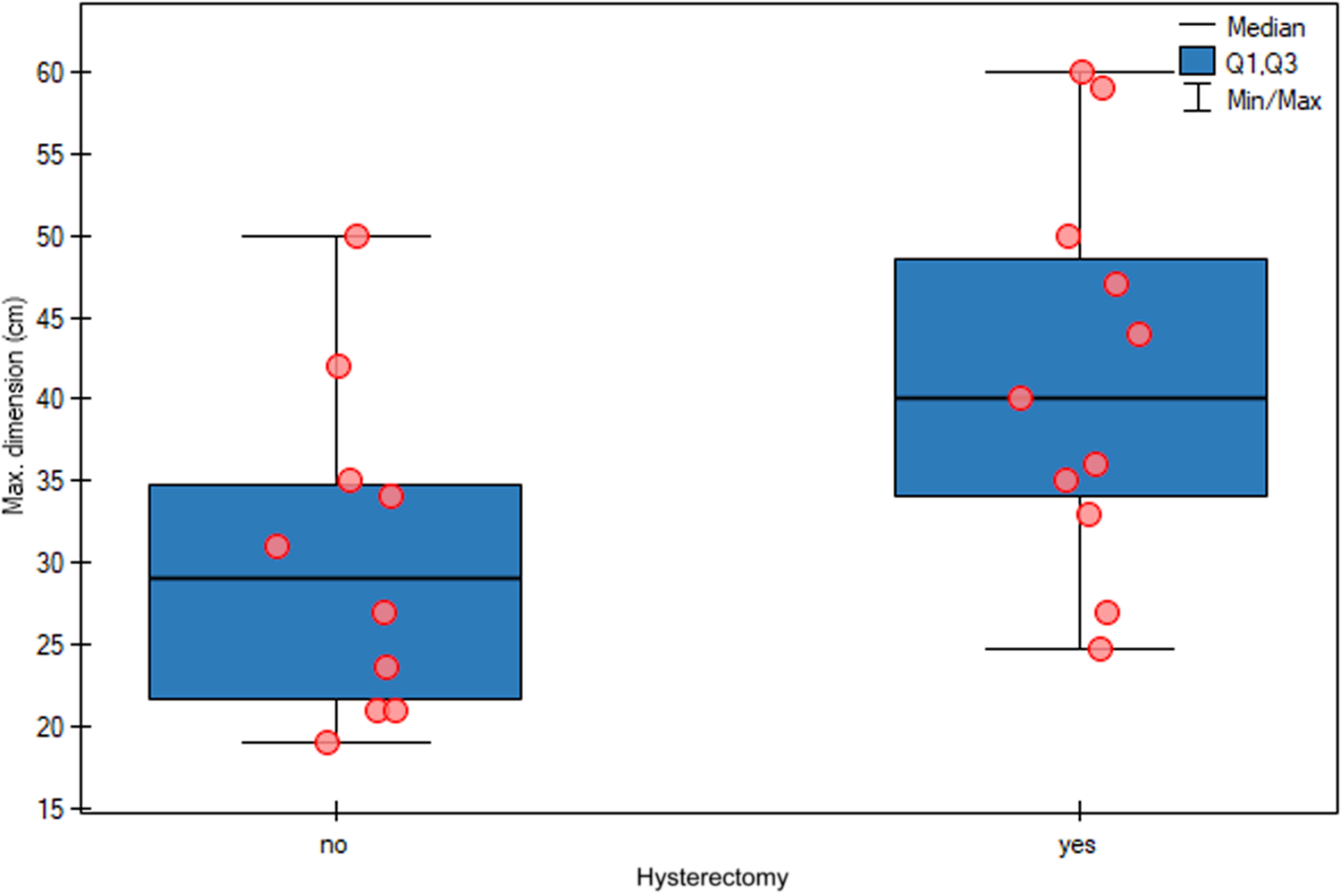
Relationship between the maximum tumour size and the decision to perform hysterectomy. Patients with larger tumours were statistically significantly more likely to undergo hysterectomy (*p* = 0.0344).

**Figure 5:**
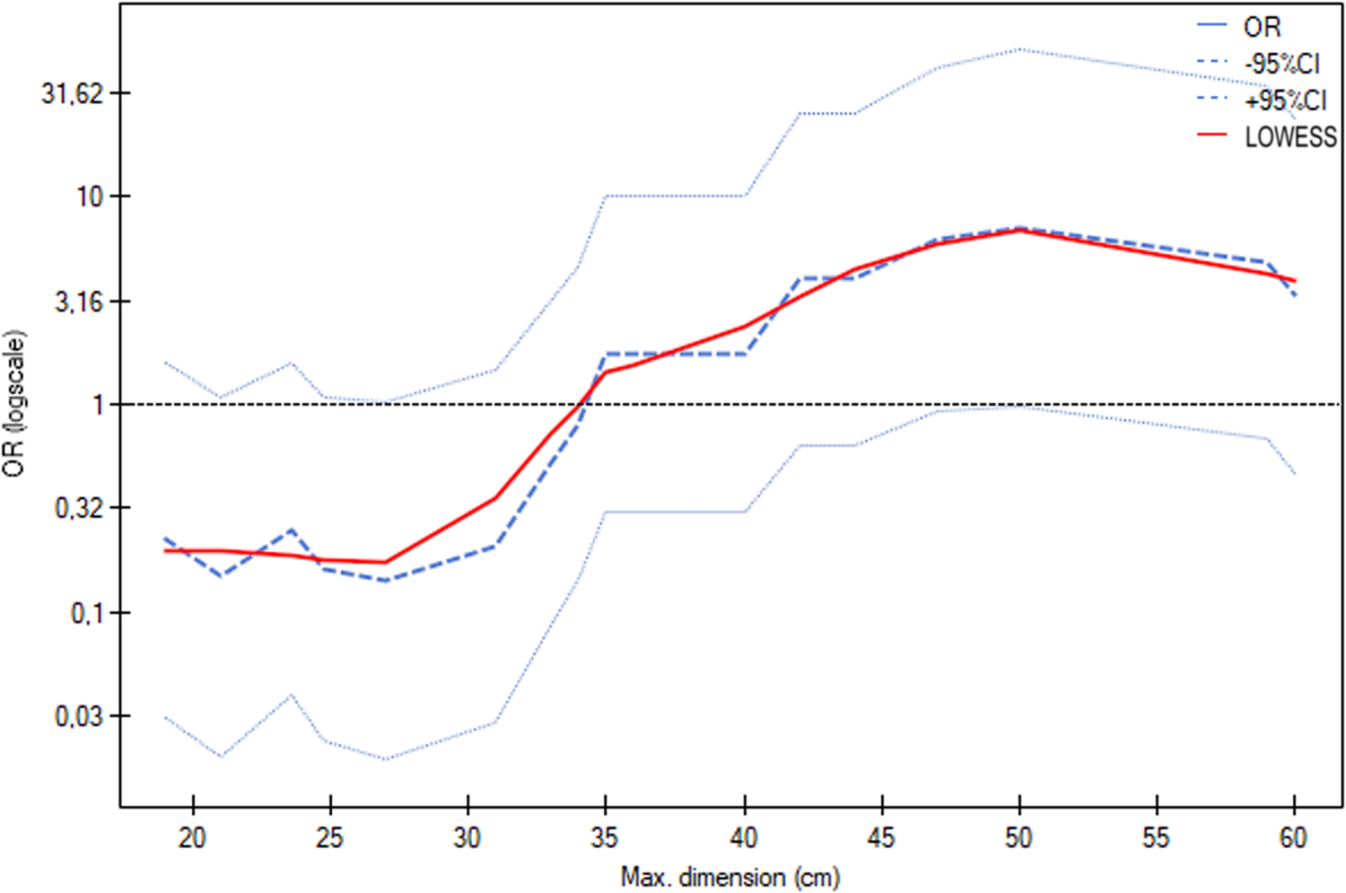
Logistic regression model for the prediction of hysterectomy based on the maximum tumour size does not indicate a significant effect of tumour size; however, the results are close to statistical significance as the odds ratio is 1.1019 with a 95% confidence interval of 0.9987 to 1.2157 (*p* = 0.0530).

The relationship between the maximum tumour size and the decision to perform hysterectomy was also examined, and patients with larger tumours were statistically significantly more likely to undergo hysterectomy (*p* = 0.0344) (Table 5 and Figure 4). The logistic regression model for the prediction of hysterectomy based on the maximum tumour size did not indicate a significant effect of tumour size; however, the results were close to statistical significance, as the odds ratio was 1.1019, with a 95% confidence interval of 0.9987–1.2157 (*p* = 0.0530) (Figure 5).

**Table 5:** Maximum tumour size in the group of patients with and without hysterectomy

		Maximum tumour size (cm)	
		Without hysterectomy	With hysterectomy
Mean		30.36	41.4273
SD		10.0977	11.8181
Median		29	40
Minimum		19	24.7
Maximum		50	60
Q1		21.65	34
Q3		34.75	48.5
Mann–Whitney U test	*Z*	2.1153	
	*p*	0.0344	

(SD – standard deviation, Q1 – first quartile, and Q3 – third quartile).

Given the limited sample and borderline statistical significance for key predictors, our logistic regression findings should be interpreted as exploratory. They highlight potential associations worthy of further study but require confirmation in larger, more robust datasets.

## 4 Discussion

In this review, we found that GBOTs were primarily unilateral and mucinous in nature and most often managed via laparotomy. Elevated CA 125 levels were more commonly seen in patients aged 40 or younger, while the likelihood of hysterectomy increased significantly in older women and those with larger tumours. Fertility-sparing surgeries were employed relatively infrequently. Below, we present the key findings from our review in the context of selected literature sources.

**Figure 6:**
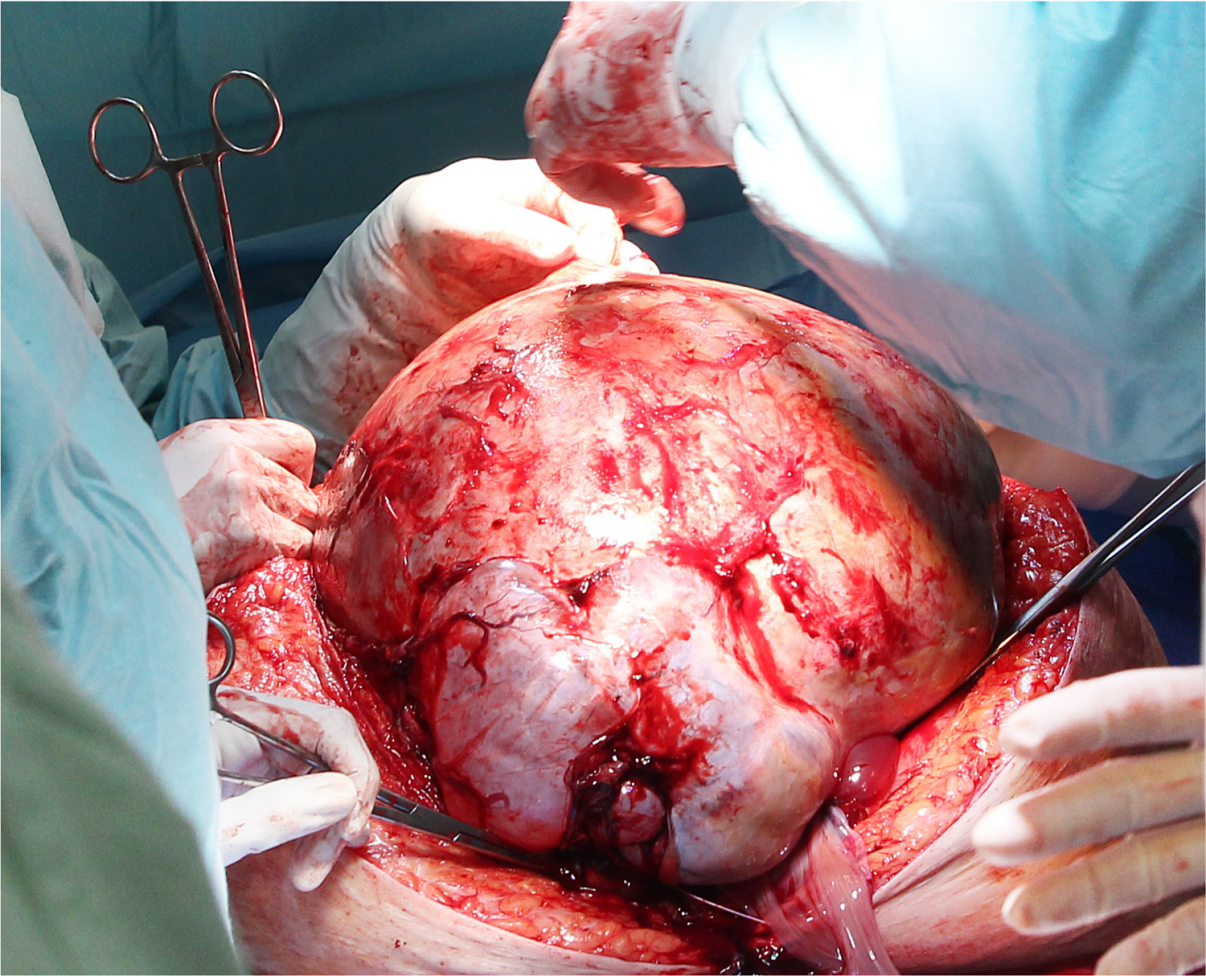
GBOT is defined as an ovarian mass with a diameter of at least 20 cm. Operative field view: A midline incision from the pubic symphysis to the xiphoid process, allowing for the safe exteriorisation of a giant ovarian tumour.

Advancements in imaging technologies and the routine implementation of screenings have greatly enhanced the early detection of ovarian masses, as they have allowed for the identification of these tumours, while they are still asymptomatic and relatively small [5]. However, in rare cases, ovarian masses may remain undetected and grow significantly, often due to limitations of transvaginal USG, patient obesity, or restricted access to medical care. There is currently no universally accepted definition for categorising large or giant ovarian tumours; generally, a GBOT is defined as an ovarian mass with a diameter of at least 20 cm (Figure 6) [6].

Mucinous tumours are typically unilateral, with bilateral cases occurring in only about 5% of the instances [7]. In the literature we analysed, all tumours were located in one of the ovaries, their average maximum dimension was 35 cm, and in terms of histopathology, the dominant type was mucinous. Ovarian cystic masses containing more than 10% borderline architecture are classified as BOTs; among these, mucinous BOTs (mBOTs) account for 45% of the cases [8]. Mucinous cystadenomas can grow into large masses and often remain undiagnosed until they reach giant sizes, typically being incidentally discovered during routine physical examinations or sonograms. Mucinous tumours are classified as benign (75%), borderline (10%), or malignant (15%); if left untreated, these tumours have the potential to progress into invasive mucinous carcinoma [1,9,10].

Based on our literature analysis, the most frequently reported symptoms in GBOTs include bloating, abdominal distension, fatigue, urinary symptoms, and pelvic or abdominal discomfort [11]. Gastrointestinal manifestations – such as abdominal pain, pressure, flatulence, and even constipation or diarrhoea – were also commonly documented, along with shortness of breath, particularly upon lying down or moving [12,13].

Diagnostics of GBOTs relies on a combination of clinical examination and advanced imaging modalities. Physical and pelvic examination may initially raise suspicion when a large adnexal mass is palpated, but definitive characterisation necessitates imaging. Transvaginal and abdominal USG serve as first-line tools: it excels at assessing lesion morphology, cystic versus solid components, septations, and distinguishing masses from ascites, thanks to its accessibility. However, its diagnostic accuracy is moderate – sensitivity around 77% and specificity roughly 83% for differentiating BOTs from benign lesions [14,15,16,17,18,19] – and limited by depth penetration in cases of giant masses. Consequently, computed tomography (CT) and magnetic resonance imaging (MRI) are recommended as supplementary modalities for comprehensive preoperative evaluation. CT provides valuable insights into tumour size, solid component characteristics, and potential metastases or lymphadenopathy and can aid in distinguishing BOTs from invasive epithelial ovarian cancers by analysing specific radiologic features such as small solid areas [15]. MRI offers superior soft-tissue contrast, enabling the detection of subtle intra-cystic nodules and papillary projections typical of borderline pathology, with MRI sensitivity and specificity reaching approximately 85 and 74%, respectively. In rare cases of GBOT, MRI and CT accurately define the lesion extent and adjacent structure involvement, guiding surgical planning [9,20].

When a GBOT is suspected, additional diagnostic tests, including tumour marker evaluations, are often conducted to assess the potential for malignancy. Suspicion of malignancy is primarily based on radiological findings and elevated levels of tumour markers, such as CA 125, human epididymis protein 4 (HE4), cancer antigen 19-9 (CA 19-9), carcinoembryonic antigen (CEA), ß-human chorionic gonadotropin (ß-hCG), α-fetoprotein (AFP), and lactate dehydrogenase [21,22]. The marker that is particularly important for distinguishing mucinous from serous tumours is CA 19-9. In some of the analysed studies, it was measured, and the levels reported in the publications were high; unfortunately, not all articles provided specific values. Another important marker for distinguishing benign from malignant lesions is HE4. When used alongside CA 125 in the risk of ovarian malignancy algorithm, it achieves a sensitivity of approximately 92% and a specificity around 75% in differentiating benign and malignant conditions [16]. In the literature we reviewed on GBOTs, HE4 was not routinely used. CA 125 is widely utilised to differentiate between malignant and benign pelvic masses, particularly in postmenopausal patients, where serum CA 125 levels above 200 U/mL have a 96% positive predictive value for malignancy [23]. However, in premenopausal patients, the specificity of CA 125 is lower due to its elevation in various benign conditions, such as endometriosis, and physiological changes in concentration during the menstrual cycle. In the publications concerning GBOTs included in our analysis, various tumour markers were used to expand preoperative diagnostics. CA 125 was measured in a majority of studies; therefore, we focused on it in our further analysis. We found a relationship close to statistical significance in patients under 40 years of age, as their CA 125 concentrations were higher compared to those in patients over 40 years. In the literature we analysed, no significant correlation between the tumour size and CA 125 levels was observed [11]. CA 125 also has a sensitivity of 50% for early-stage epithelial ovarian cancer. However, HE4 has demonstrated greater sensitivity than CA 125 in distinguishing benign from malignant conditions, making it a more effective biomarker in some cases [24]. Despite these diagnostic tools, the possibility of malignancy or borderline malignancy often remains uncertain until a final pathological diagnosis is made. In the literature we analysed, patients with mBOTs whose serum CA 125 concentrations exceeded the normal values were not qualified for surgery using minimally invasive techniques. Near-significant *p*-values must be interpreted with caution: in small samples, even moderate effect sizes may yield non-significant results, and conversely chance findings can appear significant. These small, heterogeneous samples greatly limit the external validity of findings and heighten concerns about publication bias, where positive or unusual cases are disproportionately published.

The differential diagnosis for giant ovarian cysts is extensive and covers both benign and malignant conditions of gynaecologic and non-gynaecologic origins. These can include a distended bladder, hydronephrosis, ascites, accentuated obesity, pregnancy, fibroids, and various intra-abdominal and adnexal masses [25,26,27]. Based on the cases we reviewed, the differential diagnosis commonly included benign and malignant lesions, non-epithelial tumours, and free fluid in the peritoneal cavity [28,29,30].

The management of GBOTs depends on several factors, including the patient’s age, menopausal status, fertility desires, nutritional status, access to medical facilities, and the surgeon’s expertise. Tumour size is a critical factor in determining clinical management, with surgical intervention often recommended for tumours larger than 10 cm, particularly when they cause symptoms. In cases where the tumour is particularly large, a staged surgical approach may be necessary. However, the significance of tumour size as a predictor of malignancy in ovarian tumours is still debated.

An examination of the pathological extent of BOTs is essential for appropriate intraoperative decision-making. In such cases, intraoperative frozen section analysis can help differentiate borderline tumours from invasive carcinomas, guiding the extent of surgical resection and fertility-sparing decisions. Although frozen section has moderate accuracy, sampling errors may occur, especially in large or heterogeneous tumours. Therefore, representative sampling of multiple tumour areas is crucial to minimize misdiagnosis [31]. Ultimately, integrating frozen section findings with careful gross inspection and preoperative imaging improves the likelihood of appropriate management of GBOTs.

The basic method of treating a patient with a GBOT is surgical treatment by laparotomy. This method allows for oncologically safe dissection of the tumour and its removal from the patient’s peritoneal cavity. Surgical treatment is determined based on the clinical stage of the tumour and the fertility plans of the patient. In patients with tumours confined to the ovary, corresponding to FIGO stage I, two surgical options are possible: hysterectomy with bilateral salpingo-oophorectomy can be performed if preservation of fertility is not a problem. However, if the patient wishes to preserve fertility, unilateral salpingo-oophorectomy can also be performed, leaving the ovary and uterus intact [2]. Uterine-sparing surgery in BOT patients is associated with a higher risk of recurrence, though studies show it does not increase the risk of death due to disease or death from any cause. Therefore, while uterine preservation may be considered to maintain fertility or avoid more complex surgery, women must be advised that it carries a greater likelihood of recurrence without compromising long-term survival [32]. A bilateral ovarian tumour, especially in women of childbearing age who wish to preserve fertility, presents a significant surgical challenge. In such cases, a successful two-step approach has been described in the literature, enabling accurate diagnosis of bilateral BOTs with peritoneal implants, fertility-sparing surgery, and preoperative oocyte retrieval with cryopreservation before cytoreductive intervention [33]. In the reviewed literature, more than half of the patients with GBOT underwent hysterectomy during surgery, a procedure significantly more common in those over 40 years of age and in cases with larger tumours. Moreover, hysterectomy was not performed in patients under 40 years of age.

A critical aspect of surgical treatment for malignant tumours and BOTs is ensuring complete removal of the lesion while avoiding rupture of the tumour capsule during surgery. Intraoperative rupture can lead to the intraperitoneal spread of tumour contents, which can increase the risk of disease progression and recurrence [34]. For open surgeries, it is crucial to make a sufficiently large incision and carefully free the lesion from adhesions. Adequate staging of BOTs requires meticulous inspection of the peritoneum and multiple peritoneal biopsies, while appendectomy – even in mucinous subtypes – is unnecessary unless the appendix appears macroscopically abnormal [35]. There is no supporting evidence for routine lymph node dissection in BOTs, and omission of comprehensive staging is linked to higher recurrence rates, though complete staging has not clearly demonstrated an overall survival benefit for FIGO stage I disease [36]. As hysterectomy appears to not impact survival outcomes of women with BOT, it might be avoided in the surgical staging [37].

Restaging surgery may only be justified for patients at elevated risk – such as those with serous tumours showing micropapillary features or when initial abdominal and pelvic exploration was incomplete – due to the associated morbidity and uncertain benefit. Complete removal of all visible peritoneal implants is essential both for accurate staging and therapeutic management of serous borderline ovarian tumours (sBOTs), particularly in cases exhibiting peritoneal disease, as routine lymphadenectomy has not demonstrated survival benefits in stage II/III sBOTs [36]. Fertility-sparing surgery in patients with peritoneal implants carries a higher risk of recurrence compared to stage I cases, but this elevated risk stems from the initial peritoneal spread rather than ovarian preservation itself [36].

Although laparotomy remains the predominant approach, emerging evidence supports a cautious expansion of minimally invasive techniques – especially laparoscopy – for large adnexal masses. In minimally invasive procedures, the tumour should be placed in a bag and removed through a small incision while maintaining oncological sterility [38]. Even large lesions up to 30 cm can be removed minimally invasively in a safe and effective manner. However, a cautious technique is essential: protected cyst aspiration and the use of specimen bags have been shown to significantly reduce the risk of tumour rupture during laparoscopy [39]. Similarly, five recent cases of giant cysts were managed via single-port laparoscopy with protective retrieval bags, achieving safe outcomes and excellent cosmetic results [40]. These evolving approaches – bag extraction, staged aspiration, and single-port methods – suggest that laparoscopy may be a feasible option in highly selected cases. That said, careful patient selection, meticulous technique, and oncologic safeguards remain paramount until larger prospective studies confirm the safety and long-term outcomes. An innovative approach, described by Kakinuma et al., involves the use of cyanoacrylate glue to secure the tumour within a sterile bag under laparoscopic guidance before extracting it through a small abdominal incision. This technique is particularly useful when malignancy cannot be ruled out and facilitates the removal of large tumours. However, its oncological safety in cases of BOTs would need to be confirmed through large randomised studies [41]. A similar scope of surgery in postmenopausal patients was performed in the publications we analysed. In the case of premenopausal patients, removal of the tumour alone or adnexa with the tumour was much more common. We also found that in the vast majority of cases, the decision was made to perform surgery by means of classic laparotomy with a midline incision; only in two cases it was decided to initiate treatment with minimally invasive techniques, which still required minilaparotomy to extract the tumour [42,43]. Moreover diagnostic laparoscopy – often combined with imaging (MRI, positron emission tomography [PET]-CT, and CT) and biomarkers like CA 125 and HE4 – has demonstrated high accuracy (up to 90%), with strong negative predictive value in ruling out unresectable disease and randomised trials, confirming its utility in optimising patient selection [42,43]. By enhancing preoperative assessment of intra- and extra-abdominal tumour burden, laparoscopy supports more personalised surgical planning and may safely guide decisions between primary debulking and neoadjuvant approaches [44].

The literature highlights the risk of complications in patients with giant ovarian tumours, which include life-threatening events, such as pulmonary and cardiac failure, pulmonary embolism, and sepsis [45]. These risks are heightened due to the challenges of managing massive ovarian tumours, which can cause severe hypotension, increased venous return, cardiac failure, respiratory complications, and intestinal distension. Postoperative complications often arise due to rapid changes in body circulation, including the development of pulmonary oedema [46]. One specific concern is hypotension syndrome, which is caused by the compression of large blood vessels when the patient is in a supine position; this condition can lead to sudden drops in intrathoracic and intracavitary pressure, resulting in haemodynamic disturbances. To mitigate these risks, a slow intraoperative drainage rate of 0.5–1 L/min and positioning the patient in a lateral decubitus position, rather than supine, is recommended to avoid vena cava compression and reduce the risk of cardiac arrest. In most cases reviewed in the literature, there were no intraoperative or early postoperative complications, which highlight the effectiveness of careful perioperative management. Implementation of Enhanced Recovery After Surgery (ERAS) protocols may significantly reduce the physiological stress of extensive laparotomy for ovarian tumours by promoting early feeding, mobilisation, and optimised pain control, thereby maintaining patients’ normal physiological state [47]. In patients undergoing open cytoreductive surgery for advanced ovarian masses, ERAS implementation has been associated with fewer postoperative complications and lower rates of Intensive Care Unit admission [48].

Ultimately, we found that the complexity of treating giant ovarian tumours necessitates a multidisciplinary approach, which is crucial for providing optimal patient care [49]. This approach involves collaboration among a medical oncologist, gynaecologic oncologist, radiologist, pathologist, and other specialists to develop personalised care plans. Multidisciplinary team meetings and continuous discussions of patient cases are thus essential for accurate diagnosis and determining the most effective treatment options [50,51]. The prognosis for patients with GBOTs varies based on factors such as tumour size and stage, patient age and overall health, and the success of treatment. FIGO classification is particularly important in determining the prognosis, guiding treatment decisions, and assessing outcomes [52,53].

## 5 Limitations of the study

The analysis is based on a small group of patients drawn from 21 studies, mostly of case reports and small observational studies with diverse designs and populations. Such small, heterogeneous samples severely limit the generalisability of findings, and raise concerns about publication bias – where positive or unusual cases are more likely to be reported. Although trends such as higher CA 125 levels in younger patients and an increased likelihood of hysterectomy with greater age or tumour size are intriguing, definitive conclusions require larger, prospective studies with standardised data collection protocols. An additional factor contributing to discrepancies may have been the limitation to publications in English and Polish, given that the authors were able to perform an in-depth analysis of their content.

## 6 Conclusions

GBOTs are rare neoplasms that require meticulous management to prevent high-risk operative complications. Despite the diagnostic and therapeutic challenges posed by the large size and potential complications of these tumours, with proper medical care, patients can achieve successful outcomes and good prognosis.

## Abbreviations

AFP: α-fetoprotein
BOTs: borderline ovarian tumours
BSO: bilateral salpingo-oophorectomy
CA 125: cancer antigen 125
CA 19-9: cancer antigen 19-9
CEA: carcinoembryonic antigen
CT: computed tomography
CT: computed tomography
FIGO: International Federation of Gynaecology and Obstetrics
GBOTs: giant borderline ovarian tumours
HA: abdominal hysterectomy
LDH: lactate dehydrogenase
mBOT: mucinous borderline ovarian tumour
MRI: magnetic resonance imaging
MRI: magnetic resonance imaging
PET: positron emission tomography
PRISMA: Preferred Reporting Items for Systematic Reviews and Meta-Analyses
sBOT: serous borderline ovarian tumour
ß-HCG: ß-human chorionic gonadotropin
TLH: total laparoscopic hysterectomy
USG: ultrasound
USO: unilateral salpingo-oophorectomy
